# Metagenomic analysis using next-generation sequencing of pathogens in bronchoalveolar lavage fluid from pediatric patients with respiratory failure

**DOI:** 10.1038/s41598-019-49372-x

**Published:** 2019-09-09

**Authors:** Suguru Takeuchi, Jun-ichi Kawada, Kazuhiro Horiba, Yusuke Okuno, Toshihiko Okumura, Takako Suzuki, Yuka Torii, Shinji Kawabe, Sho Wada, Takanari Ikeyama, Yoshinori Ito

**Affiliations:** 1 Department of Pediatrics, Nagoya University Graduate School of Medicine, 65 Tsurumai-cho, Showa-ku, Nagoya 466-8550 Japan; 2 Center for Advanced Medicine and Clinical Research, Nagoya University Hospital, 65 Tsurumai-cho, Showa-ku, Nagoya 466-8550 Japan; 3 Departments of Infection and Immunity, Aichi Children’s Health and Medical Center, 7-426 Morioka-machi, Obu, 474-8710 Japan; 4 Division of Pediatric Critical Care Medicine, Aichi Children’s Health and Medical Center, 7-426 Morioka-machi, Obu, 474-8710 Japan

**Keywords:** Clinical microbiology, Infectious-disease diagnostics, Viral infection, Paediatric research

## Abstract

Next-generation sequencing (NGS) has been applied in the field of infectious diseases. Bronchoalveolar lavage fluid (BALF) is considered a sterile type of specimen that is suitable for detecting pathogens of respiratory infections. The aim of this study was to comprehensively identify causative pathogens using NGS in BALF samples from immunocompetent pediatric patients with respiratory failure. Ten patients hospitalized with respiratory failure were included. BALF samples obtained in the acute phase were used to prepare DNA- and RNA-sequencing libraries. The libraries were sequenced on MiSeq, and the sequence data were analyzed using metagenome analysis tools. A mean of 2,041,216 total reads were sequenced for each library. Significant bacterial or viral sequencing reads were detected in eight of the 10 patients. Furthermore, candidate pathogens were detected in three patients in whom etiologic agents were not identified by conventional methods. The complete genome of enterovirus D68 was identified in two patients, and phylogenetic analysis suggested that both strains belong to subclade B3, which is an epidemic strain that has spread worldwide in recent years. Our results suggest that NGS can be applied for comprehensive molecular diagnostics as well as surveillance of pathogens in BALF from patients with respiratory infection.

## Introduction

In the field of infectious diseases, identification of etiologic microorganisms is essential for definitive diagnosis and decisions regarding appropriate management. Establishment of bacterial and fungal cultures is the gold standard method for identification of causative microorganisms. However, about 2–3 days are generally required to obtain quantitative culture test results, and this method is not suitable for identification of unculturable bacteria. For identification of viral pathogens, PCR and antigen tests are commonly used, but only a defined set of candidate microorganisms can be examined. Furthermore, virus isolation is a reliable method to determine the causative pathogen, but it is time-consuming, and its sensitivity may be insufficient. Although combinations of these procedures are performed, no significant pathogens are identified in 34–57% of pediatric patients^1^ and 13–62% of adult patients with pneumonia^2,3^.

Microbiological diagnosis of respiratory infection can be determined when a specific pathogen is isolated from sterile materials such as bronchoalveolar lavage fluid (BALF), transtracheal aspiration, percutaneous lung aspiration, and pleural effusion; when a significant quantity of bacteria is cultured from sputum; or when the presence of pathogens that do not usually colonize the upper respiratory tract is proven^1,4,5^. Sputum is generally used for detecting pathogens that cause lower respiratory tract infections. However, obtaining sputum is difficult, especially in pediatric patients, and distinguishing pathogens from resident bacteria in the oral cavity is sometimes difficult as well^6^. Therefore, BALF is more suitable than sputum for identifying pathogens of respiratory diseases because it can be collected from the locus of infection with less contamination with oral bacteria. BALF is often obtained for PCR of *Pneumocystis jirovecii* or cytomegalovirus (CMV) in patients with severe pneumonia^7,8^. BALF can be obtained with a relatively safe procedure and with low morbidity and mortality^9^. The diagnostic yield of causative pathogens from BALF was 28–68% in lower respiratory tract infections in immunosuppressed children including those with hematological malignancy and organ transplantation^10^. Furthermore, positive or negative results following microbiological examination of BALF lead to alteration in the management of the infection in 38.7–72.7% of patients^9–11^.

Next-generation sequencing (NGS) has been applied for comprehensive detection of causative pathogens in various infectious diseases^12–16^. We have demonstrated the utility of NGS for identification of causative or potentially causative pathogens of encephalitis, fulminant hepatitis, bloodstream infection, and acute myocarditis^17–20^. In several previous studies, NGS was applied to detect pathogens from BALF samples. Recently, Miao *et al*. have shown that significant reads of candidate pathogens were detected in 34% of BALF samples obtained from patients with infectious and noninfectious diseases^21^. However, BALF samples examined in previous studies were obtained mainly from adult patients, and no study was performed to simultaneously detect both bacteria and viruses^22–24^. In this study, we conducted comprehensive detection of pathogens from BALF samples from immunocompetent pediatric patients with severe respiratory failure using NGS.

## Results

### Comparison of DNA extraction methods for NGS

**Figure 1:**
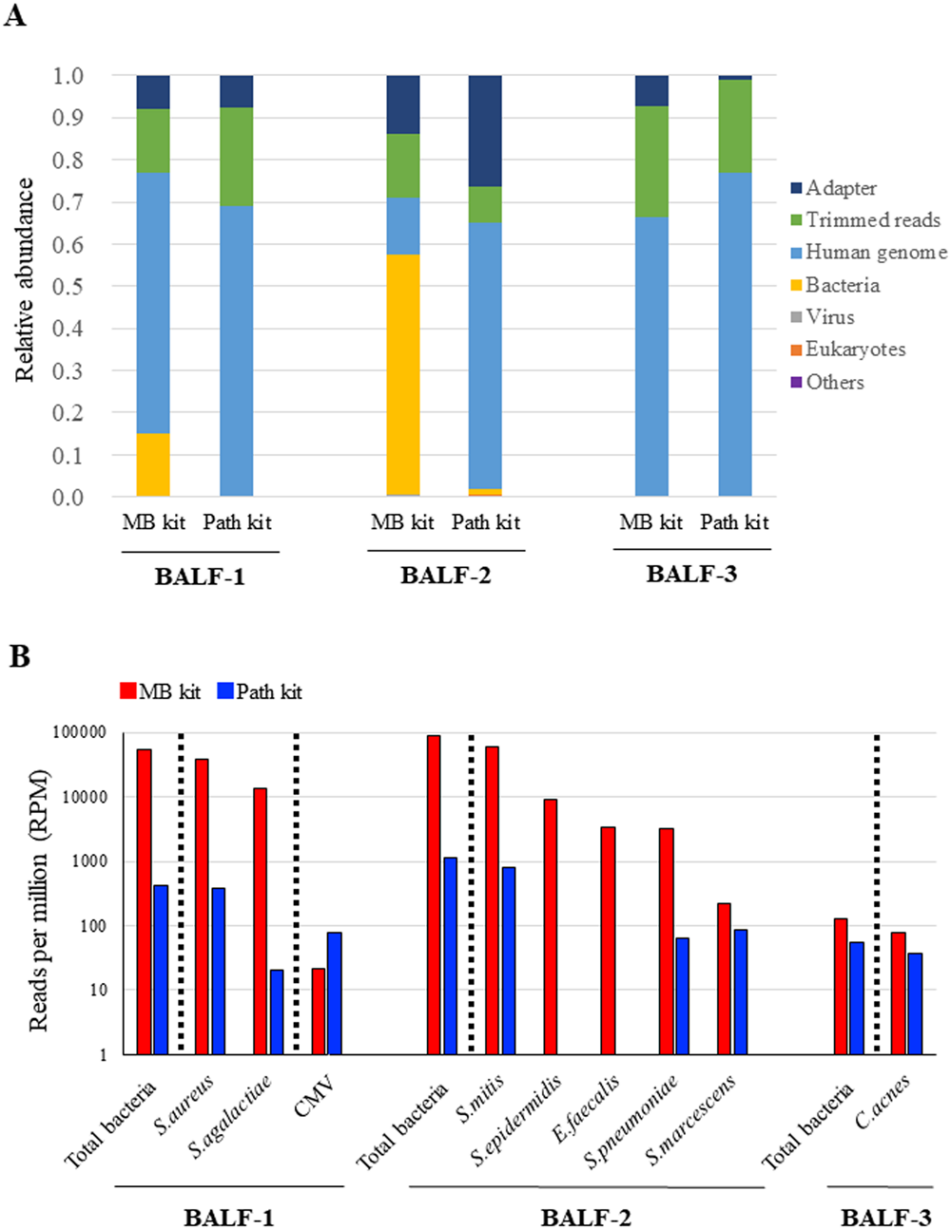
Comparison of DNA extraction kits for preparing DNA libraries to detect pathogen-derived reads. DNA libraries were prepared using DNA extracted with the QIAamp DNA Microbiome kit (MB kit) or the QIAamp UCP Pathogen Mini kit (Path kit) from bronchoalveolar lavage fluid (BALF) samples. The composition of sequence data in each DNA library was compared. Each bar represents the composition of sequence reads including adapter, trimmed reads, human genome, bacteria, virus, eukaryotes, and others (**A**). Numbers of sequencing reads assigned to each bacterial species or cytomegalovirus (CMV) are shown (**B**). RPM: reads per million.

A comparison of DNA extraction methods was performed using three BALF samples in which results of bacterial cultures and CMV PCR were available. BALF-1 was obtained from a 3-month-old boy with interstitial pneumonia. *Staphylococcus aureus* was isolated by culturing, and CMV DNA was detected with real-time PCR (2,101,786 IU/ml). BALF-2 was obtained from a 6-month-old patient with extremely low birth weight and chronic lung disease, and *Serratia marcescens* was isolated from a transtracheal aspiration sample. BALF-3 was obtained from a 7-year-old patient with pulmonary alveolar proteinosis, and no bacteria were isolated. We compared the detection efficiency of pathogen-derived reads by NGS between two types of DNA extraction kits: QIAamp DNA Microbiome kit (MB kit) and the QIAamp UCP Pathogen Mini kit (Path kit). Classifications of total reads from each library are shown in Fig. 1A. Proportions of bacterial reads were higher in libraries prepared with the MB kit than the Path kit in BALF-1 and BALF-2 (14.8% vs. 0.1% and 52.6% vs. 1.2%, respectively). Numbers of sequencing reads assigned to each bacterial species or CMV are expressed as reads per million (RPM) and shown in Fig. 1B. In BALF-1, the total number of bacterial reads obtained from the library prepared with the MB kit was more than 100 times higher than that with the Path kit. Most bacterial reads were aligned with the *S. aureus* genome in both libraries. CMV-derived reads were detectable in libraries obtained with both extraction kits. In BALF-2, approximately 80 times more total bacterial reads were obtained from the library with the MB kit compared to the Path kit. Among bacterial reads, *Streptococcus mitis*-derived reads were dominant in both libraries. Other than *S. mitis*, more than 200 RPM of four bacterial species were obtained from the library with the MB kit, whereas reads of *Staphylococcus epidermidis* and *Enterococcus faecalis* were not obtained from the library with the Path kit. *S. epidermidis and E. faecalis* were detected with real-time PCR using DNA extracted with the MB kit (1.6 × 10^5^ and 3.5 × 10^3^ copies/ml, respectively), whereas they were not detected in DNA extracted using the Path kit. In BALF-3 with a negative culture test, a small number of reads of *Cutibacterium acnes*, which is considered a member of the normal oral cavity flora, was detected in both libraries.

**Figure 2:**
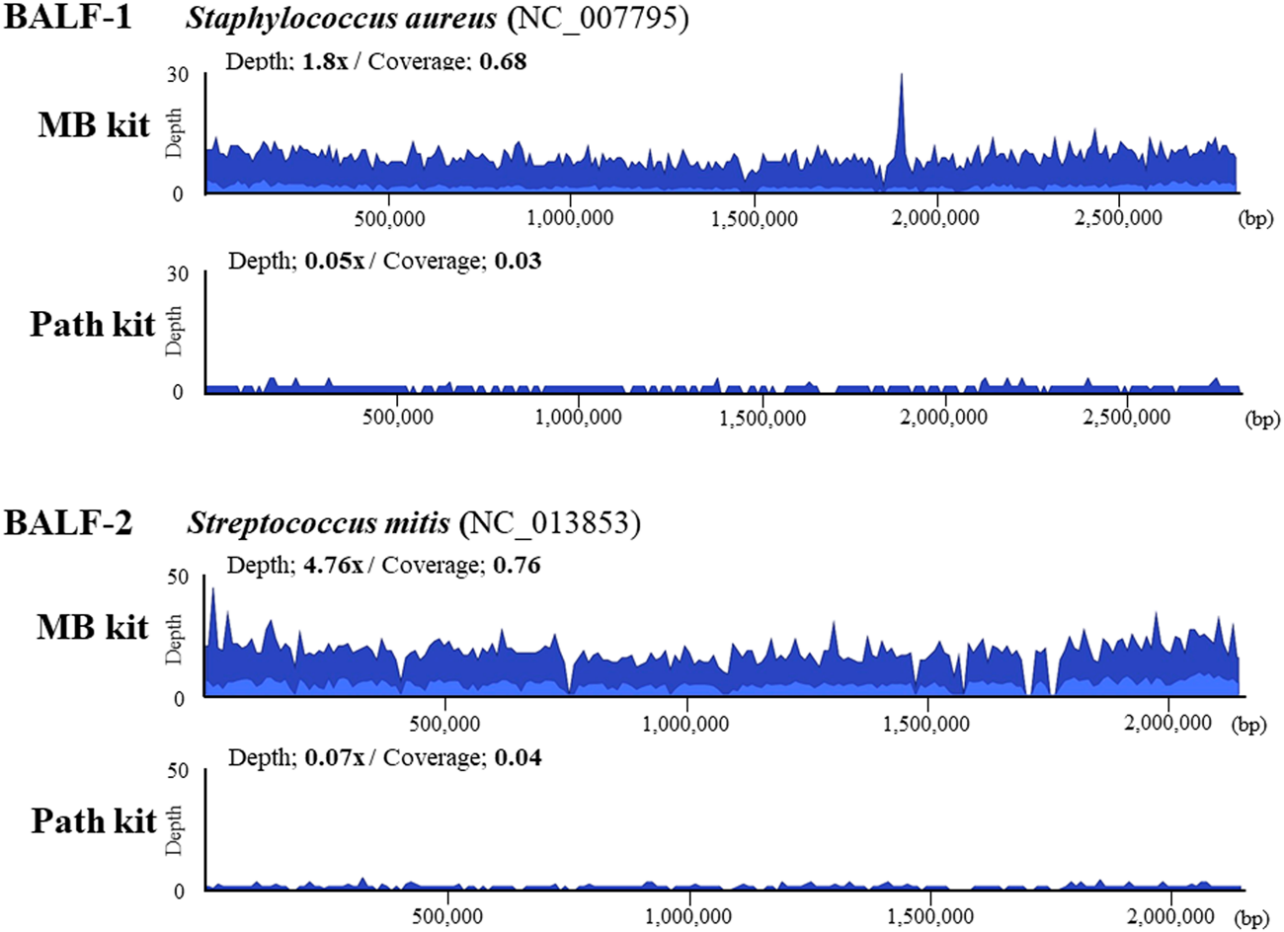
Comparison of the coverage plots of bacterial genomes. Sequencing reads detected in each bronchoalveolar lavage fluid (BALF) sample were mapped against the reference genome of *S. aureus* (NC_007795) in BALF-1 and *S. mitis* (NC_013853) in BALF-2. DNA for library preparation was extracted using the QIAamp DNA Microbiome kit (MB kit) or the QIAamp UCP Pathogen Mini kit (Path kit). Blue and dark blue colors in the bacterial genome alignment represent average and maximal coverage in the aggregated 1-kbp region, respectively.

The comparison of the read mapping results is shown in Fig. 2. The average coverage (depth) and the fraction of reference covered (coverage) of sequenced reads mapped to the reference genome of *S. aureus* (NC_007795) or *S. mitis* (NC_013853) were much higher in libraries prepared with the MB kit than those with the Path kit. Taken together, these results indicate that the MB kit is more suitable than the Path kit for preparing a DNA library to detect bacterial reads in BALF samples. Considering that the difference in CMV reads was small in BALF-1, the MB kit was used in further experiments.

### Comprehensive detection of pathogen-derived sequences from BALF samples from pediatric patients with severe respiratory failure

**Figure 3:**
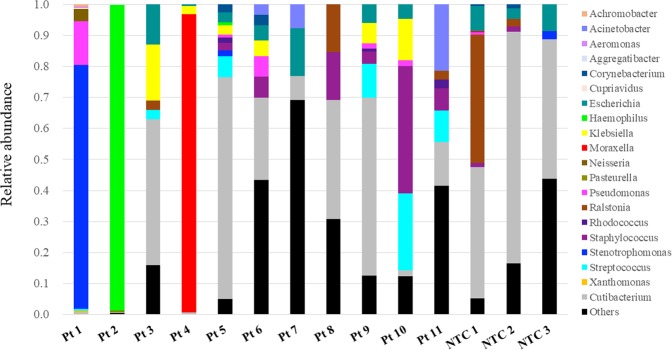
Genus composition of BALF samples from patients with severe respiratory failure. Each bar represents taxa at the genus level of taxonomic hierarchy. Bacterial reads with fewer than 10 reads or without annotations at the genus level were classified as “Others”. Pt 1-10 and NTC 1-3 indicate libraries prepared from bronchoalveolar lavage fluid (BALF) and distilled water, respectively.

**Figure 4:**
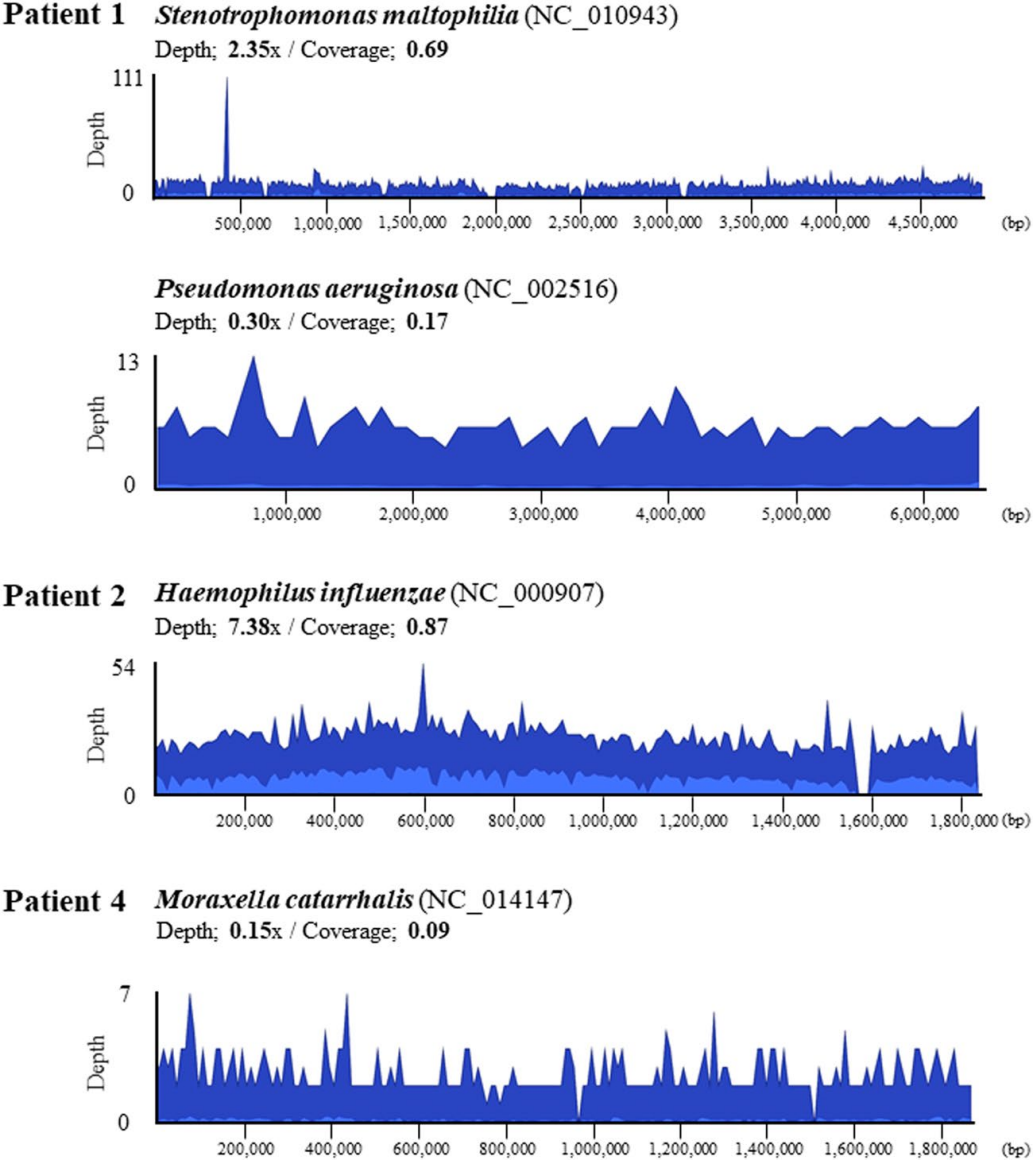
Coverage plots of bacterial genomes detected in patients with severe respiratory failure. Sequencing reads detected in bronchoalveolar lavage fluids of each patient were mapped to the reference genome of *S. maltophilia*, *P. aeruginosa*, *H. influenzae*, and *M. catarrhalis*. Blue and dark blue colors in the bacterial genome alignment represent average and maximal coverage in the aggregated 1-kbp region, respectively.

We investigated 10 BALF samples from pediatric patients with severe respiratory failure (patients 1–10) using NGS to identify etiologic agents. In addition, one patient in the chronic phase of idiopathic interstitial pneumonia was included as a control (patient 11). Patient characteristics are listed in Table 1. A mean of 2,041,216 total reads was sequenced for each library. The classification of the sequence data is shown in Supplementary Table 1. To avoid making calls based on spurious alignments or cross-contamination, we considered bacterial or viral reads as significant when the number of sequences aligned to each reference genome was above 10 RPM, and the sequenced reads covered wide ranges of the reference genome with the read mapping approach. Composition of bacterial reads at the genus level of each DNA-sequencing library is shown in Fig. 3, and a summary of the pathogens with significant bacterial and viral reads is listed in Table 2. A significant number of four types of bacterial reads was detected in three BALF samples with DNA-sequencing (patients 1, 2, and 4), and NGS results for three bacteria were consistent with the results of transtracheal aspiration culture. Sequencing reads of bacteria identified with DNA-sequencing in these three patients were also detected with RNA-sequencing (data not shown). Substantial bacterial reads of *Cutibacterium* and *Rastonia*, which are considered contamination, were detected in libraries from distilled water (Fig. 3 and Supplementary Table 1). Sequence coverage and depth of each reference bacterial genome are shown in Fig. 4. In patient 1, *Stenotrophomonas maltophilia* and *Pseudomonas aeruginosa* reads were detected with NGS, whereas only the latter was isolated in a small amount with culturing. Conversely, no significant bacterial read was identified in patient 5 or 6, in which *Streptococcus pneumoniae* and *Haemophilus influenzae*, respectively, were isolated with culturing of transtracheal aspiration.

**Table 1:** Patient characteristics.

Pt No.	Age	Sex	Underlying disease	Respiratory failure	Circulatory failure	X-ray findings of pneumonia	ICU LOS (days)	Mechanical ventilation (days)	ECMO	Outcome
1	5y 9m	F	Cerebral palsy^a^	+	+	+	12	7	−	Recovered
2	28d	M	none	+	+	−	14	5	−	Recovered
3	0y 11m	F	TFO, Bronchomalacia	+	+	−	10	6	+	Recovered
4	0y 4m	F	21 trisomy	+	−	−	8	4	−	Recovered
5	16d	M	none	+	+	−	9	4	−	Recovered
6	0y 3m	F	none	+	+	−	13	9	−	Recovered
7	4y 11m	M	none	+	−	−	7	6	−	Recovered
8	8y 10m	F	none	+	−	+	8	6	−	Recovered
9	0y 1m	F	none	+	+	+	59	52	+	Recovered
10	7y 0m	F	none	+	+	−	15	12	+	Recovered
11	2y 5m	F	IIPs^b^	−	−	−	−	−	−	−

Abbreviation: ECMO, extracorporeal membrane oxygenation; ICU, intensive care unit; IIPs, idiopathic interstitial pneumonias; LOS, length of stay. TFO, Tetralogy for Fallot. ^a^A patient with cerebral palsy after bacterial meningitis. She had undergone ventriculoperitoneal shunt placement procedure for hydrocephalus. ^b^A patient with chronic phase of IIPs used for negative control.

**Table 2:** Sequence results using NGS for detection of pathogens from BALF samples.

Pt No.	NGS results								Conventional method results		
	DNA-sequencing				RNA-sequencing				Bacterial culture	Viral antigen	PCR
	Total reads	Detected pathogen	Number of reads	RPM	Total reads	Detected pathogen	Number of reads	RPM			
1	2,603,238	***S. maltophilia***	100,083	38,446	2,303,752	**HMPV**	322	140	*P. aeruginosa* (±)	HMPV	—
		***P. aerginosa***	16,947	6,510							
2	2,631,210	***H. influenzae***	103,738	39,426	2,937,810	**HRSV-A**	342	116	*H. influenzae* (2+)	HRSV	HRSV-A
3	2,071,836	—	—	—	2,676,318	**HRV-B**	50,138	18,734	—	—	HRV
4	2,754,852	***M. catarrhalis***	2,428	881	2,946,090	—	—	—	*M. catarrhalis* (2+)	—	—
5	3,608,962	—	—	—	2,402,562	**HRSV-A**	1,612	671	*S. pneumoniae* (±)	—	HRSV-A
6	1,489,176	—	—	—	1,685,054	**HRSV-B**	83,212	49,382	*H. influenzae* (2+)	HRSV	HRSV-B
7	1,660,692	—	—	—	1,531,292	**EV-D68**	115,343	75,324	—	—	—
8	1,419,054	—	—	—	1,549,278	**EV-D68**	784,380	506,287	—	—	—
9	1,373,122	—	—	—	1,478,618	—	—	—	—	—	CMV
10	1,581,982	—	—	—	1,517,564	—	—	—	—	HMPV	—
11^a^	1,252,872	—	—	—	1,431,422	—	—	—	—	—	—

Abbreviation: CMV, cytomegalovirus; EV-D68, enterovirus D-68; HMPV, human metapneumovirus; HRSV, human respiratory syncytial virus; HRV, human rhinovirus; Pt, patient; RPM, read per million. ^a^A patient with chronic phase of idiopathic interstitial pneumonias used for negative control.

**Figure 5:**
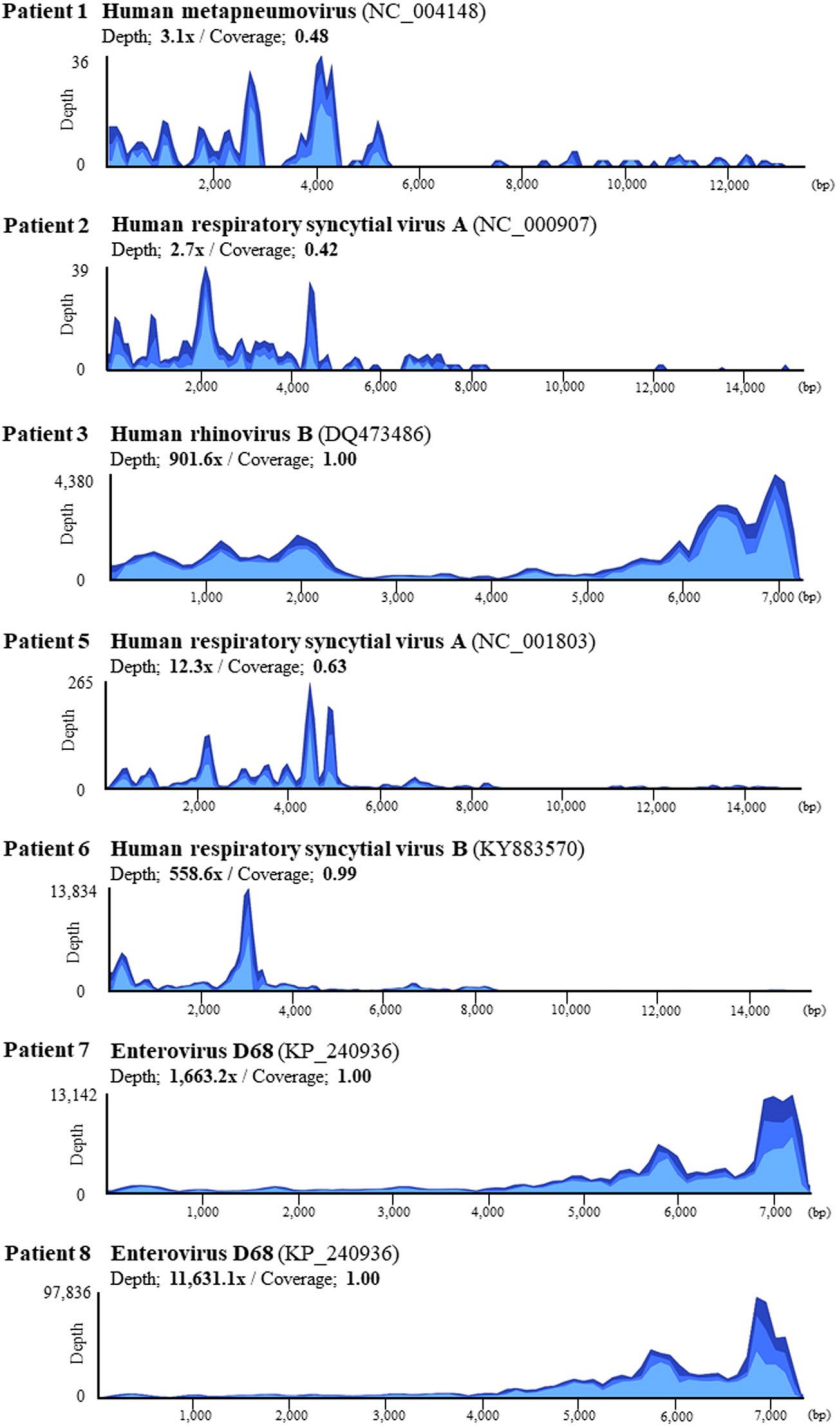
Coverage plots of viral genomes detected in patients with severe respiratory failure. Sequencing reads detected in bronchoalveolar lavage fluids of each patient were mapped to the reference genome of human metapneumovirus (HMPV), human respiratory syncytial virus (HRSV)-A, human rhinovirus (HRV)-B, HRSV-B, and enterovirus (EV)-D68. Light blue, blue, and dark blue colors in the viral genome alignment represent minimal, average, and maximal coverage in the aggregated 10-bp region, respectively.

**Figure 6:**
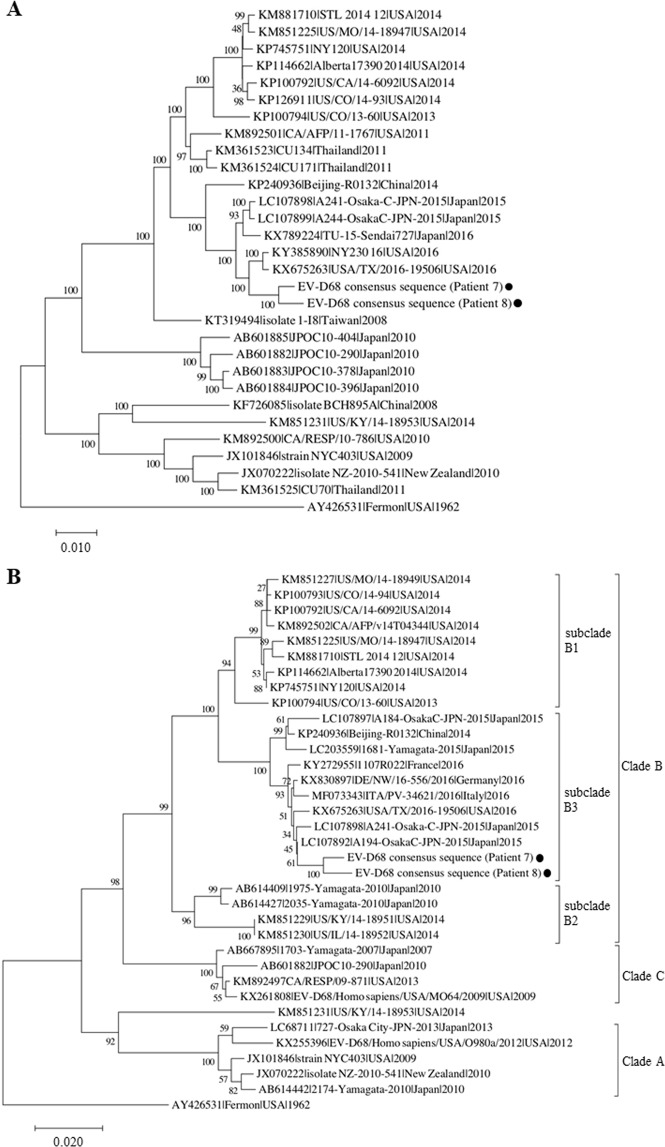
Phylogenetic analysis of enterovirus D68. Phylogenetic trees of full genomes (**A**) and VP1 sequences (**B**) of enterovirus D68 (EV-D68) consensus sequences obtained from patients 7 and 8 are shown. The phylogenetic relationships of the registered sequences of EV-D68 were estimated using the neighbor-joining method with 1,000 replicates using MEGA7^43^. The percentage of replicate trees in which the associated taxa clustered together in the bootstrap test is shown next to the branches. The tree is drawn to scale, with branch lengths in the same units as those of the evolutionary distances used to infer the phylogenetic tree. The evolutionary distances were computed using the p-distance method and are in units of the number of base differences per site. Clades were defined previously based on VP1 sequences^37,50,51^. GenBank accession number, strain name, country of origin, and year of detection are shown for each strain. Scale bars show the genetic distance.

Candidate pathogenic respiratory viruses were detected in seven of 10 patients with RNA-sequencing: human respiratory syncytial virus (HRSV) from three patients, enterovirus D68 (EV-D68) from two patients, and human metapneumovirus (HMPV) and human rhinovirus B (HRV-B) from one patient each. Viral antigen test results were confirmed by retrospective review of medical records, and most were consistent with NGS results. Additionally, we performed multiplex RT-PCR to confirm the presence of the viral genome that was identified with NGS, except EV-D68. As a result, the NGS-based approach for detection of the causative virus was consistent with the results of multiplex virus PCR except in patient 1 in whom HMPV was not confirmed with PCR. Sequence coverage and depth of each reference viral genome are shown in Fig. 5. In patients 3, 6, 7, and 8, read mapping to each reference genome was achieved with high coverage and depth, and almost the complete viral genome was obtained. Phylogenetic analysis of the EV-D68 full genome suggested that the EV-D68 sequences derived from patients 7 and 8 were genetically related to the EV-D68 viruses circulating worldwide in 2016 (Fig. 6A). Based on phylogenetic analysis of the VP1 region, the EV-D68 strains detected in this study belong to subclade B3 (Fig. 6B). Among the two NGS-negative cases in which neither bacteria nor viruses were detected, CMV and HMPV were detected with PCR and an antigen test, respectively (patients 9 and 10). No significant bacterial or viral reads were identified from the negative control BALF sample (Patient 11). Consensus sequences of detected viruses with high coverage (HRV-B from patient 3, HRSV-B from patient 6, and EV-D68 from patients 7 and 8) are deposited in the DNA Data bank of Japan (Accession numbers: LC495296, LC495297, LC495298, and LC495299).

## Discussion

In this study, we used NGS to perform comprehensive detection of pathogenic microorganisms from BALF samples from 10 pediatric patients with severe respiratory failure. We expected that all microorganisms including viruses could be detected by conducting both DNA- and RNA-sequencing; indeed, a significant number of bacterial or viral sequencing reads was detected in eight of 10 patients. Furthermore, candidate causative pathogens of respiratory failure were detected in three cases (patients 3, 7, 8) in which pathogenic microorganisms were not identified by conventional methods. To the best of our knowledge, this is the first study to use NGS to comprehensively investigate both bacteria and viruses from BALF specimens in pediatric patients with respiratory failure.

In the present study, we first verified two DNA extraction kits to improve efficiency in bacteria detection from BALF samples, and showed that the QIAamp DNA Microbiome kit was more suitable. The QIAamp DNA Microbiome kit is a “microbial enrichment” DNA extraction kit. After the human cell lysis step, exposed nucleic acids are degraded with Benzonase nuclease, whereas the bacterial cells theoretically remain intact. In this experiment, this microbial enrichment procedure resulted in an approximately 100-fold increase in the number of bacterial reads compared with the other DNA extraction kit.

A discrepancy in the results between NGS and the culture test was observed in some cases. In patient 1, the number of reads annotated to *S. maltophilia* was much higher than that of *P. aeruginosa*, which was the only bacterial strain isolated from aspirated sputum by culturing. In contrast, no significant bacterial read of isolated bacteria was identified with NGS in patient 5 or 6. Bacterial culturing with samples from these patients was performed using transtracheal aspirated sputum obtained on the same day as BALF. Therefore, the discrepancy between NGS and the culture test results may reflect the difference in the specimens, and some of the isolated bacteria from aspirated sputum could be colonizing bacteria in the upper respiratory tract. Unfortunately, because the culture test using BALF was not performed in some patients, a comparison of the results of NGS and culturing was difficult. Furthermore, sufficient amounts of BALF were not available in some cases, which may have resulted in a false negative NGS-based result. On the other hand, a discrepancy between NGS and the bacterial culture results of BALF or other specimens was observed in a previous study^21^.

In this study, a significant number of viral reads was detected in seven of 10 patients. Among them, reads of more than 10,000 RPM were annotated to each virus reference genome in four patients, suggesting that BALF contains abundant reads of pathogenic viruses for respiratory infections. Because NGS does not require virus-specific primers, the NGS-based approach is especially useful for detection of RNA viruses, which have much higher mutation rates than DNA viruses^16^. The pathogenicity of the detected viruses has already been established in immunocompetent pediatric patients^25–30^. In general, HRV is considered to have relatively weak virulence, although it sometimes causes severe respiratory disease, especially in patients with immunodeficiency or anatomical abnormalities of the airway^30^. HRV is classified into three subtypes, HRV-A, -B, and -C, based on gene sequence analysis. The detection rate of HRV-B is lower than the other subtypes. Whether the severity of clinical symptoms is related to HRV subtypes remains inconclusive^30–32^. Patient 3, in whom HRV-B was detected, had congenital tracheomalacia as an underlying disease, suggesting that HRV-B may have been a causative pathogen or trigger of respiratory failure.

EV-D68 was identified in two patients without underlying diseases (patients 7 and 8). EV-D68 can cause severe lower respiratory illness and asthma exacerbation, mostly in children^29,33^. EV-D68 infections are more likely to be associated with severe and life-threating respiratory diseases than other enterovirus genotypes^29^. In 2014, a large-scale outbreak of severe respiratory infection caused by EV-D68 was reported in the US and other countries^29,33^. In Japan, EV-D68 outbreaks coincided with spikes in acute asthma exacerbations and acute flaccid myelitis in 2015^34^, and another epidemic of EV-D68 was reported in 2018^35^. Phylogenetic analysis suggested that the EV-D68 sequences identified in this experiment belong to subclade B3 and are related to epidemic strains that spread worldwide in 2016^36,37^. These results indicate that the NGS-based approach enables us not only to comprehensively detect the causative virus, but also to specify genotypes of the detected virus in a single test, which would contribute to molecular epidemiological studies. In patient 1, HMPV was detected with NGS and the antigen test, but it was not detected with PCR. The primer sequences may not have been appropriate for detection of the HMPV strain in this patient.

Co-infection with bacteria and viruses was observed in four of 10 patients. Using NGS, *S. maltophilia*, *P. aeruginosa*, and HMPV were detected in patient 1, and *H. influenzae* and HRSV were detected in patient 2. HRSV was identified with NGS in patients 5 and 6, whereas *S. pneumoniae* and *H. influenzae* were isolated in patient 5 and patient 6, respectively, by culturing. Although concurrent bacterial infection in infants with HRSV or HMPV infection is relatively uncommon, co-infection may worsen the clinical symptoms of virus infection^28,38^. Therefore, respiratory failure may have been induced by co-infection with bacteria and the respiratory viruses in these patients. Conversely, because viral shedding of HRSV may continue for as long as 3 to 4 weeks, especially in young children^39^, detection of a small number of HRSV reads may reflect its presence as a bystander.

In our study, neither a significant number of bacterial reads nor viral reads were detected in two patients by NGS. Patient 10 was diagnosed with fulminant myocarditis with respiratory failure. In this patient, an antigen test using a nasopharyngeal swab sample was HMPV positive, whereas HMPV was not detected in BALF, neither with NGS nor PCR. Although HMPV may be a trigger of development of fulminant myocarditis, a false-positive result of the HMPV antigen test should be considered. In patient 9, a discrepancy between NGS and CMV PCR results was observed. Insufficient host genome digestion may have decreased the sensitivity of detection of CMV DNA because the concentration of extracted DNA was relatively higher than that of other specimens. Another reason for the discrepancy may be that CMV particles that are latent in human cells were simultaneously digested with Benzonase nuclease at the time of DNA extraction. Considering the above, more sequence depth may be required to detect DNA viruses with NGS.

Some limitations of our NGS-based approach for detecting pathogens should be discussed. First, the microbial enrichment procedure increased the number of bacterial reads, but still, 92.9–99.6% of trimmed NGS reads were derived from human DNA. More efficient methods to remove host nucleic acids are desirable to improve the sensitivity of detecting pathogen-derived genomes and to reduce the cost of the NGS procedure. Second, NGS can identify fungi in clinical samples^21^, but we could not validate whether our NGS procedure could detect fungal DNA. Third, we considered more than 10 RPM of bacterial or viral reads as significant in this study. However, determining a threshold read count was difficult. Furthermore, detection of viral or bacterial reads with NGS does not always indicate the presence of viable or pathogenic microorganisms. Finally, we utilized MePIC v2.0 (National Institute of Infectious Disease, Tokyo, Japan)^40^ as a metagenomic pathogen identification tool in this study. This pipeline incorporates the MEGABLAST program for a homology search against known nucleotide sequences registered in the NCBI nt database. Therefore, microorganisms whose sequences are unknown or not registered in the database cannot be detected. In addition, reproducibility issues in bioinformatics may occur due to several reasons such as the short half-life of the bioinformatics software, the complexity of the pipelines, and the incompleteness in workflow description^41^.

In conclusion, we demonstrated the utility of the NGS-based approach for detection of pathogens in BALF from pediatric patients with severe respiratory failure. Although further improvement of NGS workflow may be required, NGS can be applied for molecular diagnostics as well as surveillance of pathogens in the field of infectious diseases.

## Methods

### Ethics statement

This study was performed in compliance with relevant laws and institutional guidelines and was approved by the Institutional Review Board of Nagoya University Graduate School of Medicine and Aichi Children’s Health and Medical Center. Written informed consent was obtained from all patients or their legal guardians.

### Patients and samples

BALF samples were obtained from 10 pediatric patients hospitalized in the pediatric intensive care unit with respiratory failure and investigated with NGS for comprehensive detection of pathogens. No patients had a history of an immunocompromised state. Patient characteristics are listed in Table 1. BALF was obtained during the acute phase of the illness and cryopreserved at −80 °C until use. Until the NGS analysis was completed, the researchers who conducted experiments and analyses were not notified of the medical information of each patient or of the results of conventional microorganism tests including cultures, viral antigen tests, and PCR.

### Library preparation and sequencing

First, we compared two DNA extraction kits: the QIAamp DNA Microbiome kit (Qiagen, Hilden, Germany) and the QIAamp UCP Pathogen Mini kit (Qiagen) for efficacy of detection of bacterial reads. Based on the results, the QIAamp DNA Microbiome kit was used in further experiments. RNA extraction was performed using the NucleoSpin RNA Blood kit (MACHEREY-NAGEL, Düren, Germany). The extracted RNA was immediately converted to cDNA and amplified with the REPLI-g WTA Single Cell kit (Qiagen) in accordance with the manufacturer’s instructions. DNA and RNA concentrations were measured using a Qubit dsDNA HS assay kit (Thermo Fisher Scientific, Waltham, MA, USA) and a Qubit RNA HS assay kit (Thermo Fisher Scientific), respectively.

The Nextera XT DNA Sample Preparation Kit (Illumina, San Diego, CA, USA) was used to prepare all the libraries from extracted DNA or generated cDNA, as described above. Libraries were also prepared from distilled water as a preparation control (NTC 1-3). Library quality was analyzed using an Agilent 2200 TapeStation (Agilent Technologies, Santa Clara, CA, USA) or Agilent 2100 Bioanalyzer (Agilent Technologies). The library concentration was quantified using a Qubit dsDNA HS assay kit (Thermo Fisher Scientific). Then, libraries were sequenced on MiSeq (Illumina) with the 2 × 150 bp paired-end protocol.

### Processing of sequence data

For analysis of the sequence data, the FASTQ files were uploaded to and processed with MePIC v2.0 (National Institute of Infectious Disease)^40^. First, unnecessary adapter sequences and low-quality bases (Q-score cutoff, 20) were trimmed off in the pipeline. Then, human-derived reads were detected using the BWA program and removed from the downstream analysis. For the remaining reads, the MEGABLAST program (E-value cutoff, 1e–30) was used to search similar sequences of known nucleotide sequences registered in the NCBI nt database. Finally, the search result was downloaded and summarized regarding taxonomic information using MEGAN6 (University of Tübingen, Tübingen, Germany) (bit score >250 (bacteria)/>50 (virus))^42^. The raw sequence data in FASTQ format was also used for alignment with the reference genome with CLC Genomics Workbench 9.5 (CLC bio; Qiagen) (length fraction = 0.9 (bacteria)/0.8 (virus); similarity fraction = 0.9 (bacteria)/0.8 (virus); nonspecific reads were ignored).

Full-length consensus sequences of EV-D68 were obtained by assigning the most common nucleotide sequence gained in this study to each nucleotide position of the reference genome (KP240936). Phylogenetic analysis of the full genome and the VP1 region of EV-D68 was conducted using the neighbor joining method by MEGA7^43^.

### Real-time PCR

Real-time PCR for detection of CMV was performed with the QuantiTect Multiplex PCR kit (Qiagen), and amplification was conducted using the QuantStudio^TM^3 Real-Time PCR System (Applied Biosystems, Foster City, CA, USA) as previously described^44^. To confirm the presence of viruses detected by NGS, reverse transcription PCR (RT-PCR) was performed with the Cycleave PCR respiratory virus detection kit (Takara Bio, Kusatsu, Japan), and amplification was conducted using the QuantStudio^TM^3 Real-Time PCR System in accordance with the manufacturer’s instructions. Real-time PCR for *S. epidermidis* and *E. faecalis* was performed by a commercial laboratory (TechnoSuruga Laboratory, Shizuoka, Japan) using Rotor-Gene (Qiagen). Information about the primers and probes used for PCR is shown in Supplementary Table 2^44–49^.

## Supplementary information

Supplementary Tables.

## Data Availability

Consensus sequences of detected viruses are available in the DNA Data bank of Japan (Accession numbers: LC495296, LC495297, LC495298, and LC495299). All other data generated or analyzed during this study are included in this article.
