# Supplementary material for: Metagenomic analysis using next-generation sequencing of pathogens in bronchoalveolar lavage fluid from pediatric patients with respiratory failure
Source: Sci Rep. 2019 Sep 9;9:12909. doi: 10.1038/s41598-019-49372-x (PMC6733840; doi:10.1038/s41598-019-49372-x)
Supplement: Supplementary file 1 — Supplementary Tables. [file 41598_2019_49372_MOESM1_ESM.docx]

**Supplementary Information**

**Metagenomic analysis using next-generation sequencing of pathogens in bronchoalveolar lavage fluid from pediatric patients with respiratory failure**

Suguru Takeuchi^1^, Jun-ichi Kawada^1^, Kazuhiro Horiba^1^, Yusuke Okuno^2^, Toshihiko Okumura^1^, Takako Suzuki^1^, Yuka Torii^1^, Shinji Kawabe^3^, Sho Wada^4^, Takanari Ikeyama^4^, and Yoshinori Ito^1^

^1^ Department of Pediatrics, Nagoya University Graduate School of Medicine, 65 Tsurumai-cho, Showa-ku, Nagoya 466-8550 Japan.

^2^ Center for Advanced Medicine and Clinical Research, Nagoya University Hospital, 65 Tsurumai-cho, Showa-ku, Nagoya 466-8550 Japan.

^3^ Departments of Infection and Immunity, Aichi Children’s Health and Medical Center, 7-426 Morioka-machi, Obu 474-8710 Japan.

^4^ Division of Pediatric Critical Care Medicine, Aichi Children’s Health and Medical Center, 7-426 Morioka-machi, Obu 474-8710 Japan.

**Corresponding author:** Jun-ichi Kawada, M.D., Ph.D.

Department of Pediatrics, Nagoya University Graduate School of Medicine

65 Tsurumai-cho, Showa-ku, Nagoya 466-8550, Japan

Tel: +81-52-744-2294; Fax: +81-52-744-2974

E-mail: [kawadaj@med.nagoya-u.ac.jp](mailto:kawadaj@med.nagoya-u.ac.jp)

| **Supplementary Table 1. Compositions of sequence data** | | | | | | | | | | | |
| --- | --- | --- | --- | --- | --- | --- | --- | --- | --- | --- | --- |
|  | Pt No. | Total reads | Adapter (%) | Trimmed  reads (%) | Human  genome (%) | Blast hits (%) |  | Compositions of Blast hits | | | |
|  |  |  |  |  |  |  |  | Eukaryotes | Bacteria | Viruses | Others |
| DNA-seq | 1 | 2,603,238 | 332,144 (13) | 867,753 (33) | 1,303,696 (50) | 57,259 (2) |  | 1,902 | 55,275 | 61 | 21 |
|  | 2 | 2,631,210 | 160,541 (6) | 911,185 (35) | 1,472,983 (56) | 66,882 (3) |  | 2,367 | 64,507 | 2 | 6 |
|  | 3 | 2,071,836 | 196,016 (9) | 822,829 (40) | 1,045,966 (50) | 865 (0) |  | 754 | 106 | 0 | 5 |
|  | 4 | 2,754,852 | 260,689 (9) | 802,043 (29) | 1,680,681 (61) | 3,078 (0) |  | 1,799 | 1,262 | 14 | 3 |
|  | 5 | 3,608,962 | 375,353 (10) | 983,643 (27) | 2,233,171 (62) | 4,275 (0) |  | 3,169 | 1,099 | 0 | 7 |
|  | 6 | 1,489,176 | 196,504 (13) | 499,430 (34) | 788,580 (53) | 649 (0) |  | 579 | 65 | 5 | 0 |
|  | 7 | 1,660,692 | 175,955 (11) | 645,338 (39) | 834,222 (50) | 951 (0) |  | 681 | 12 | 8 | 1 |
|  | 8 | 1,419,054 | 165,950 (12) | 461,720 (33) | 787,278 (55) | 792 (0) |  | 921 | 13 | 13 | 4 |
|  | 9 | 1,373,122 | 73,242 (5) | 509,434 (37) | 786,727 (57) | 818 (0) |  | 588 | 189 | 13 | 2 |
|  | 10 | 1,581,982 | 126,902 (8) | 565,925 (36) | 885,188 (56) | 702 (0) |  | 690 | 105 | 18 | 5 |
|  | 11 | 1,252,872 | 265,267 (21) | 403,690 (32) | 578,998 (46) | 587 (0) |  | 501 | 72 | 8 | 6 |
| RNA-seq | 1 | 2,303,752 | 164,447 (7) | 743,868 (32) | 1,332,877 (58) | 19,650 (1) |  | 809 | 18,218 | 435 | 188 |
|  | 2 | 2,937,810 | 177,503 (6) | 1,135,071 (39) | 1,509,801 (51) | 48,471 (2) |  | 1,317 | 44,175 | 162 | 2,783 |
|  | 3 | 2,676,318 | 157,080 (6) | 740,837 (28) | 1,682,920 (63) | 44,726 (2) |  | 1,132 | 8,232 | 30,786 | 4,576 |
|  | 4 | 2,946,090 | 186,981 (6) | 899,856 (31) | 1,668,741 (57) | 84,557 (3) |  | 4,107 | 58,329 | 104 | 22,017 |
|  | 5 | 2,402,562 | 155,485 (6) | 770,016 (32) | 1,436,995 (60) | 3,900 (0) |  | 1,141 | 1,601 | 752 | 406 |
|  | 6 | 1,685,054 | 154,211 (9) | 640,816 (38) | 778,683 (46) | 45,999 (3) |  | 414 | 5,437 | 38,232 | 1,916 |
|  | 7 | 1,531,292 | 144,405 (9) | 581,326 (38) | 694,681 (45) | 51,138 (3) |  | 452 | 542 | 49,946 | 198 |
|  | 8 | 1,549,278 | 131,943 (9) | 517,469 (33) | 247,556 (16) | 384,113 (25) |  | 245 | 8,047 | 372,346 | 3,475 |
|  | 9 | 1,478,618 | 148,183 (10) | 509,164 (34) | 755,142 (51) | 17,256 (1) |  | 609 | 12,167 | 24 | 4,456 |
|  | 10 | 1,517,564 | 124,536 (8) | 557,031 (37) | 809,897 (53) | 1,039 (0) |  | 352 | 551 | 11 | 125 |
|  | 11 | 1,431,422 | 136,969 (10) | 531,426 (37) | 583,456 (41) | 78,248 (5) |  | 1,277 | 59,513 | 25 | 17,433 |
| NTC | 1 | 68,580 | 58 (0) | 22,818 (33) | 39,682 (58) | 2,868 (4) |  | 190 | 2649 | 21 | 8 |
|  | 2 | 7,104 | 15 (0) | 1,119 (16) | 2,375 (33) | 1,070 (15) |  | 70 | 991 | 8 | 1 |
|  | 3 | 5,874 | 56 (1) | 892 (15) | 672 (11) | 473 (8) |  | 26 | 436 | 5 | 6 |

The numbers in the table indicate the number of reads of each component analyzed using MePIC.

NTC: Preparation control libraries that were generated from distilled water instead of input DNA.

| **Supplementary Table 2. Primer and probe sequences used for PCR** | | |  |
| --- | --- | --- | --- |
| Virus | Primer/probe | Sequence (5'→3') |  |
| CMV^44^ | Forward primer | GACTAGTGTGATGCTGGCCAAG |  |
|  | Reverse primer | GCTACAATAGCCTCTTCCTCATCTG |  |
|  | Probe | AGCCTGAGGTTATCAGTGTAATGAAGCGCC |  |
| HRSV-A^45^ | Forward primer | AACAGATGTAAGCAGCTCCGTTATC |  |
|  | Reverse primer | CGATTTTTATTGGATGCTGTACATTT |  |
|  | Probe | TGCCATAGCATGACACAATGGCTCCT |  |
| HRSV-B^46^ | Forward primer | AACAGACATAAGCAGCTCAGTAATT |  |
|  | Reverse primer | CGATTTTTGTTGGATGCAGTGCATTT |  |
|  | Probe | GCGAGCAGGAGCTATAGTGTCATGCTATGGTAGCTCGC |  |
| HPIV-1^47^ | Forward primer | ACCTACAAGGCAACAACATC |  |
|  | Reverse primer | CTTCCTGCTGGTGTGTTAAT |  |
|  | Probe | GCTGCC CAAACGATGGCTGAAAAAGGGA GGCAGC |  |
| HPIV-2^47^ | Forward primer | CCATTTACCTAAGTGATGGAA |  |
|  | Reverse primer | CGTGGCATAATCTTCTTTTT |  |
|  | Probe | GCTGCCAATCGCAAAAGCTGTTCAGTCACGGCAGC |  |
| HPIV-3^47^ | Forward primer | GGAGCATTGTGTCATCTGTC |  |
|  | Reverse primer | TAGTGTGTAATGCAGCTCGT |  |
|  | Probe | CGCGCTACCCAGTCATAACTTACTCAACAGCAACAGCGCG |  |
| HMPV^48^ | Forward primer | AACCGTGTACTAAGTGATGCACTC |  |
|  | Reverse primer | CATTGTTTGACCGGCCCCATAA |  |
|  | Probe | CTTTGCCATACTCAATGAACAAAC |  |
| Flu A^47^ | Forward primer | AAAGCGAATTTCAGTGTGAT |  |
|  | Reverse primer | GAAGGCAATGGTGAGATTT |  |
|  | Probe | GCTGCCAGGGCTTTCACCGAAGAGGGGGCAGC |  |
| Flu B^47^ | Forward primer | GTCCATCAAGCTCCAGTTTT |  |
|  | Reverse primer | TCTTCTTACAGCTTGCTTGC |  |
|  | Probe | GCTGCCAACGAAGTAGGTGGAGACGGAGGGGCAGC |  |
| AdV^46^ | Forward primer | CCCTGGTAKCCRATRTTGTA |  |
|  | Reverse primer | AATGACAGGCTGYTGAGYC |  |
|  | Probe | GCTGCCAACCAGTCYTTGGTCATGTTRCATTGGGCAGC |  |
| HBoV^46^ | Forward primer | GAAAGACAAGCATCGCTCC |  |
|  | Reverse primer | TGGGTGTTCCTGATGATATG |  |
|  | Probe | CGCGCTGGAGCAGGAGCCGCAGCCCGATAGCGCG |  |
| HRV^49^ | Forward primer | GACARGGTGTGAAGSYCGACARGGTGTGAAGSYC |  |
|  | Reverse primer | CAAAGTAGTYGGTCCCATCC |  |
|  | Probe | TCCTCCGGCCCCTGAATGYGGCTAA |  |
| Abbreviation: AdV, adenovirus; CMV, cytomegalovirus; Flu, influenza virus; HBoV, human bocavirus; HPIV, human parainfluenza virus; HRSV, human respiratory syncytial virus; HRV, human rhinovirus. | | | |
|  | | |  |
